# Dehydrozingerone, a Curcumin Analog, as a Potential Anti-Prostate Cancer Inhibitor In Vitro and In Vivo

**DOI:** 10.3390/molecules25122737

**Published:** 2020-06-12

**Authors:** Sariya Mapoung, Shugo Suzuki, Satoshi Fuji, Aya Naiki-Ito, Hiroyuki Kato, Supachai Yodkeeree, Natee Sakorn, Chitchamai Ovatlarnporn, Satoru Takahashi, Pornngarm Limtrakul (Dejkriengkraikul)

**Affiliations:** 1 Department of Biochemistry, Faculty of Medicine, Chiang Mai University, Chiang Mai 5200, Thailand; srmapoung@gmail.com (S.M.); yodkeelee@hotmail.com (S.Y.); 2 Center for Research and Development of Natural Products for Health, Chiang Mai University, Chiang Mai 5200, Thailand; 3 Department of Experimental Pathology and Tumor Biology, Nagoya City University Graduate School of Medical Sciences, Nagoya 467-8601, Japan; suzuki.shugo@med.osaka-cu.ac.jp (S.S.); sfuji777gm2013@gmail.com (S.F.); ayaito@med.nagoya-cu.ac.jp (A.N.-I.); h.kato@med.nagoya-cu.ac.jp (H.K.); sattak@med.nagoya-cu.ac.jp (S.T.); 4 Department of Pharmaceutical Chemistry, Faculty of Pharmaceutical Sciences, Prince of Songkla University, Hat Yai, Songkhla 90110, Thailand; natee.sa@mail.wu.ac.th (N.S.); lchitcha@pharmacy.psu.ac.th (C.O.); 5 Drug Delivery System Excellence Center, Prince of Songkla University, Hat-Yai, Songkhla 90110, Thailand

**Keywords:** curcumin analogs, dehydrozingerone, prostate cancer, pharmacokinetic, anticancer

## Abstract

Curcumin (Cur) exhibits biological activities that support its candidacy for cancer treatment. However, there are limitations to its pharmacological effects, such as poor solubility and bioavailability. Notably, the use of Cur analogs has potential for addressing these limitations. Dehydrozingerone (DZG) is a representative of the half-chemical structure of Cur, and many reports have indicated that it is anticancer in vitro. We, therefore, have hypothesized that DZG could inhibit prostate cancer progression both in vitro and in vivo. Results revealed that DZG decreased cell proliferation of rat castration-resistant prostate cancer, PLS10 cells, via induction of the cell cycle arrest in the G1 phase in vitro. In the PLS10 xenograft model, DZG significantly decreased the growth of subcutaneous tumors when compared to the control via the inhibition of cell proliferation and angiogenesis. To prove that DZG could improve the limitations of Cur, an in vivo pharmacokinetic was determined. DZG was detected in the serum at higher concentrations and remained up to 3 h after intraperitoneal injections, which was longer than Cur. DZG also showed superior in vivo tissue distribution than Cur. The results suggest that DZG could be a candidate of the Cur analog that can potentially exert anticancer capabilities in vivo and thereby improve its bioavailability.

## 1. Introduction

Prostate cancer is the second cause of cancer-related deaths among men worldwide [1,2]. The survival rate for prostate cancer patients depends on the stage of the disease and the point of diagnosis [3]. The five-year survival rate of early-stage prostate cancer patients is more than 99%, while in the advanced metastatic stage, it is only 28% [3]. The growth of prostate cancer cells is relevant to the androgen level; therefore, androgen deprivation therapy (ADT), a treatment to reduce androgen levels and/or block androgen receptor activity, has been introduced to most early-stage prostate cancer patients [4]. Although prostate cancer patients respond positively to ADT, two to three years after initiating treatment, castration-resistant prostate cancer (CRPC) can develop. CRPC is not only resistant to ADT or chemotherapeutic treatment, but it is also highly metastatic, which can result in tumor cells becoming more aggressive [5]. The expected median period of survival for recurrent prostate cancer patients is only 12–15 months, even with the administration of chemotherapies [6]. Therefore, a novel treatment for advanced prostate cancer has been of significant interest to a number of researchers.

**Figure 1:**
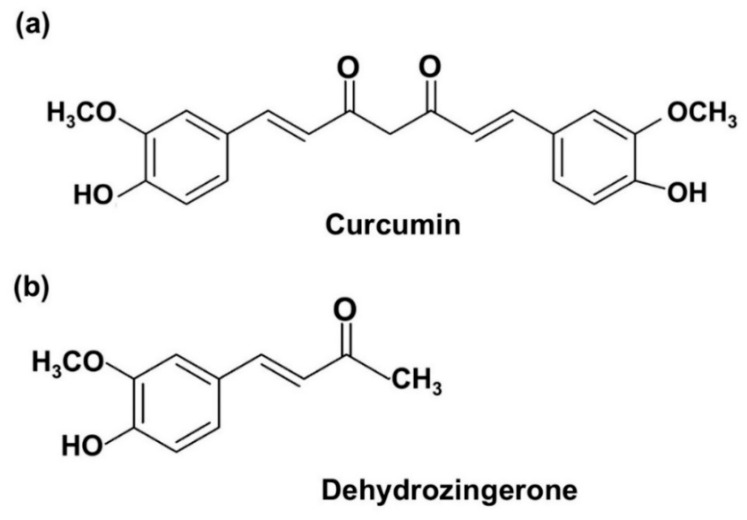
Chemical structure of curcumin (Cur) (**a**) and dehydrozingerone (DZG) (**b**).

Curcumin (Cur) (Figure 1a), a yellow pigment found in the rhizome of the plant *Curcuma longa Linn*., has been extensively used as a food coloring agent in various products such as curry powder. It has served as an active component in many Ayurvedic, Chinese, Hindu and Thai traditional medicines used for the treatment of a number of diseases. Cur has long been intensively studied, and it was found to display interesting therapeutic efficacies against various human diseases, such as cancer, diabetics, inflammation and cardiovascular diseases [7]. Importantly, Cur was found to be safe for human use. Subjects who were given high doses of 12 g/day exhibited poor bioavailability [8]. However, its clinical applications were obstructed due to some of its own limitations. Poor absorption, rapid metabolism and rapid systematic elimination are the major causes of low plasma levels and tissue distribution. Various approaches have been used to improve its pharmacokinetic profiles, such as by decreasing Cur metabolism via adjuvant therapies or by co-administering it along with metabolic relevant inhibitors. The key enzyme in Cur metabolism is glucuronosyltransferase. Therefore, the co-administration of Cur and certain glucuronosyltransferase inhibitors, such as piperine, quercetin and silibinin, might improve the pharmacokinetic properties [9]. The second approach involves using nanotechnology to formulate Cur-nanoparticles such as Cur-liposome and polylactic-co-glycolic acid-encapsulated Cur [10,11]. Third, the chemical structure modification of Cur has been used to improve its biological activity and bioavailability. Since the ß-diketone motif of Cur might be the cause of instability and rapid metabolism, the structural modification or removal of the β-diketone motif could improve its solubility, stability and bioavailability, as well as its other relevant biological activities [12].The monocarbonyl analogs of Cur (MAC) represent one group of Cur analogs that are capable of removing the β-diketone moiety. A previous study reported that MAC improves the stability and solubility of Cur in vitro [12].

**Figure 2:**
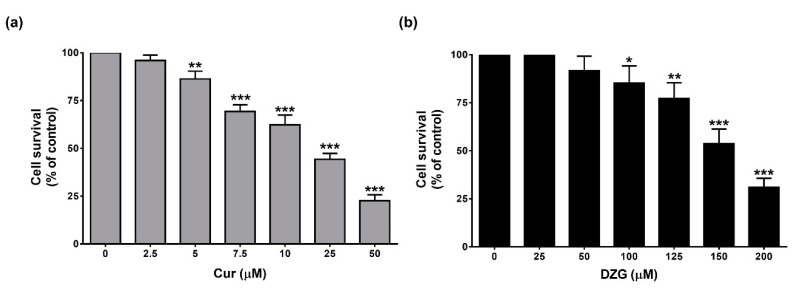
Cytotoxicity of Cur (**a**) and DZG (**b**) on PLS10 cells. Cells were treated with Cur (0–50 μM) or DZG (0–200 µM) and incubated for 48 h. Cell viability was measured by WST-1 assay. Data are represented as mean ± S.D. of three independent experiments. * *p* < 0.05, ** *p* < 0.01 and *** *p* < 0.001 vs. control (one-way ANOVA, Dunnett’s multiple comparisons test, tested groups vs. control group).

**Figure 3:**
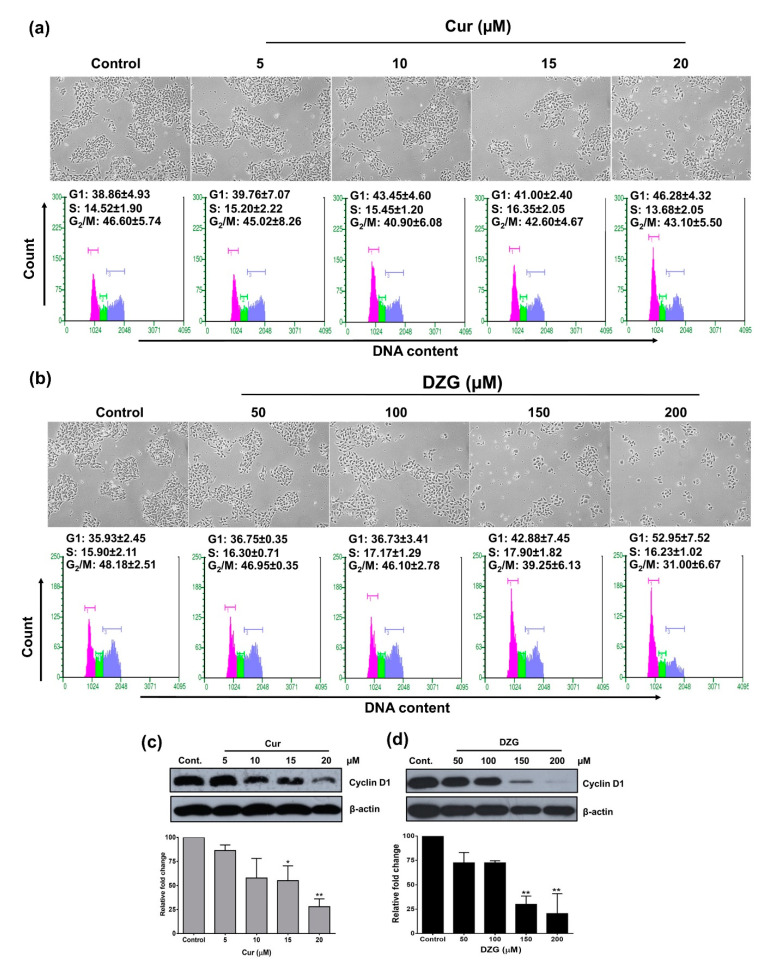
Cur and DZG treatment induced G1 phase cell cycle arrest in PLS10 cells. Cells were treated with Cur (0–20 μM) and DZG (0–200 μM) for 48 h. Cells were harvested for analysis of the cell cycle distribution by the Guava cycle (**a**,**b**), while cyclin D1 expression was determined by Western blot analysis (**c**,**d**). The level of protein expression was quantified by IMAGE J software. Data are presented as mean ± S.D. of three independent experiments. * *p* < 0.05 and ** *p* < 0.01 vs. control (one-way ANOVA, Dunnett’s multiple comparisons test, tested groups vs. control group).

**Figure 4:**
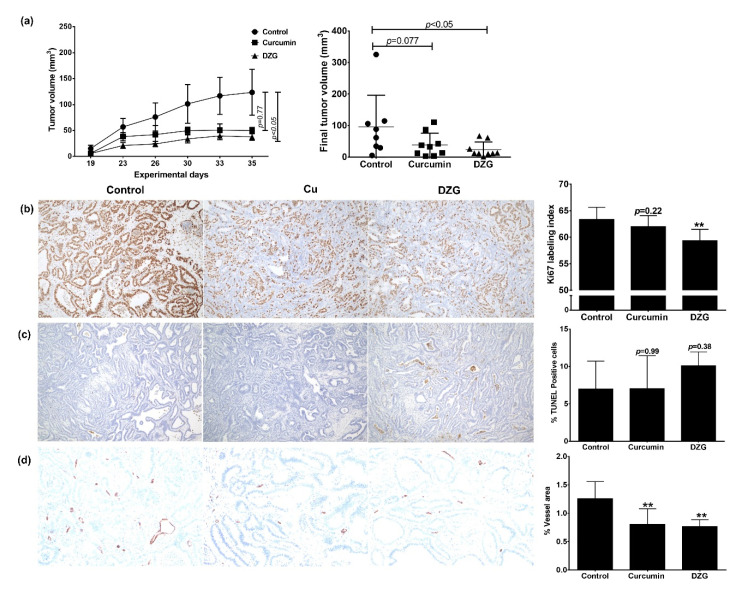
DZG inhibited cell proliferation and angiogenesis in vivo using PLS10 cells xenograft model. Mice were subcutaneously injected with PLS10 cells and treated two times per week with Cur or DZG (30 mg/kg body weight) for a period of 5 weeks. Tumor volume was measured twice a week and at the end of the study (**a**). The tumor masses were collected, sectioned and analyzed by immunohistochemistry to detect the Ki67-labeling index (**b**), % TUNEL (Terminal deoxynucleotidyl transferase dUTP nick end labeling)-positive cells (**c**) and % of the vessel area (**d**). Data presented in bar diagrams represent the mean ± S.E of eight mice in the control and nine mice in the treatment groups. ** *p* < 0.01 vs. control (one-way ANOVA, Dunnett’s multiple comparisons test, tested groups vs. control group).

Dehydrozingerone (DZG, Figure 1b), (*E*)-4-(4-hydroxy-3-methoxyphenyl)-but-3-en-2-one, can be obtained from ginger (*Zingiber officinale* Roscoe, family Zingiberaceae) and through a laboratory-based synthesis procedure [13]. DZG, known as a feruloylmethane analog of Cur, is recognized as a degradant that contains half of the structure of Cur [5]. DZG shares many structural similarities with Cur but lacks the β-diketone moiety; therefore, DZG could overcome the limitations of Cur in ways that have been described above. DZG possesses antioxidant and anti-inflammatory activities that are similar to those of Cur, but only a few studies have been conducted on the anticancer properties of DZG [13,14]. A previous study revealed that DZG could inhibit 12-*O*-tetradecanoylphorbol-13-acetate (TPA)-induced Epstein-Barr virus early antigen (EBV-EA) activation in EBV genome-carrying human lymphoblastoid cells in a similar manner to Cur [15]. In addition, DZG effectively inhibited human colon cancer, HT-29 cells proliferation via induction of the cell cycle arrest and the accumulation of intracellular ROS [16]. Even though DZG has been found to display anticancer activities based on a number of in vitro studies, its in vivo anticancer, pharmacokinetic and tissue distributions have not been reported elsewhere. In this report, we investigated the effects of DZG on cancer progression in vitro against rat castration-resistant prostate cancer, PLS10 cells and in vivo using the PLS10 cells xenograft model. Additionally, the in vivo pharmacokinetic and tissue distributions of DZG in the mice models were also determined.

## 2. Results

### 2.1. DZG Inhibited Cell Proliferation of Castration-Resistant Prostate Cancer, PLS10 Cells

The antiproliferative effects of Cur and DZG on PLS10 cells in vitro were determined by WST-1 assay. The results revealed that both Cur and DZG significantly (*p* < 0.001) inhibited cell proliferation in a dose-dependent manner (Figure 2a,b). The IC_50_ of Cur and DZG were 20.33 ± 0.58 µM and 153.13 ± 11.79 μM, respectively. These results suggest that Cur displayed stronger cell proliferation inhibitory effects than DZG in vitro.

### 2.2. DZG Induced Cell Cycle Arrest in G1 Phase

The results indicated that the cell population in the G1 phase slightly increased from 38% in the control group to 46% in 20 μM of the Cur treatment (Figure 3a). While the G1 phase population in the DZG treatment at the concentrations 150 and 200 µM dramatically increased from 36% in the control group to 43% and 53%, respectively (Figure 3b). In addition, the G2/M cell population decreased from 48% in the control to 39% and 31% in 150 and 200 µM of the DZG-treated groups, respectively (Figure 3b). The high concentrations of Cur (15 and 20 µM) and DZG (150 and 200 µM) treatments decreased in terms of cyclin D1 expression (*p* < 0.01) in PLS10 cells (Figure 3c,d). The results above indicate that both Cur and DZG inhibited PLS10 cells growth via induction of the cell cycle arrest in the G1 phase by the downregulation of cyclin D1.

### 2.3. In Vivo Antitumor Activity

After the treatment of 30 mg/kg body weight of Cur or DZG via intraperitoneal (i.p.) injection two times a week for five weeks on male BALB/c-nu/nu mice, the survival percentage of nude mice in each groups were 100% (data not shown). The body weight and organ weight, liver and kidneys of mice in Cur and DZG-treated groups were not found to be statistically different from the control group (Table 1). This result suggests that Cur and DZG have no toxicity in mice and could be safe for clinical trial applications. As a result, the tumor volume significantly decreased from 96 cm^3^ in the control group to 24 cm^3^ (*p* < 0.05) in the DZG-treated group, whereas, at the same dose, Cur did not significantly decrease the tumor volume (tumor volume was 38 cm^3^; *p* = 0.77) (Figure 4a). To analyze the antitumor effects of Cur and DZG, cell proliferation and apoptosis in tumor sections were determined. The results showed that the Ki67-labeling index significantly decreased from 63 in the control group to 59 (*p* < 0.01) in the DZG-treated group (Figure 4b), while the Cur-treated group reported similar results to the control group (Ki67-labeling index was 62%; *p* = 0.223). However, the apoptotic cells increased from 7% in the control group to 10% in the DZG treatment group, but the results were not statistically significant (*p* = 0.38), whereas the Cur treatment group showed similar results (*p* = 0.99) to the control group (Figure 4c). For the detection of angiogenesis in tumors, the number of CD31-positive areas was significantly reduced in both the Cur and DZG-treated groups when compared to the control group (*p* < 0.05) (Figure 4d). According to these experiments, it could be summarized that DZG exhibited in vivo anticancer capabilities via the inhibition of cancer cell proliferation and angiogenesis. These data suggest that the effects of DZG were more potent than those of Cur on in vivo tumor growth with the inhibition of tumor proliferation and angiogenesis.

### 2.4. In vivo Pharmacokinetics and Tissue Distribution

**Figure 5:**
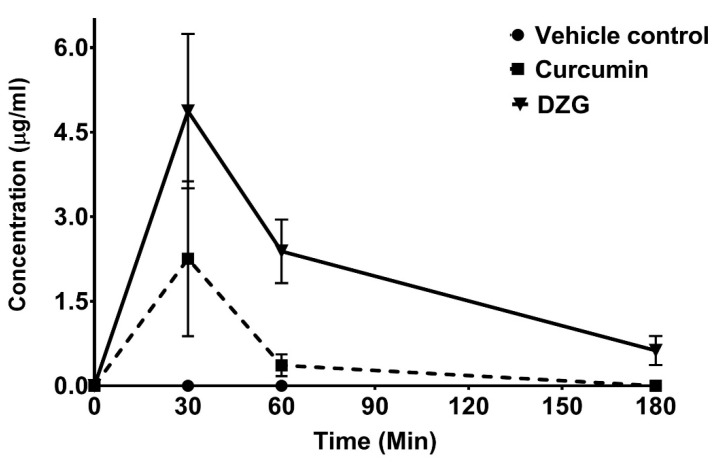
Levels of serum Cur and DZG recorded in mice after receiving a single dose of Cur or DZG (100 mg/kg body weight) by intraperitoneal (i.p.) injection. Serum concentrations of Cur and DZG were determined by high-performance liquid chromatography (HPLC) using a reversed-phase C18 column (Waters). The mobile phase values were 52% acetonitrile and 48% citric buffer. Each point represents the mean ± S.E. of three or four mice.

The concentrations of Cur and DZG in the serum and tissue at each time point were evaluated by high-performance liquid chromatography (HPLC). The vesicle control was 50% DMSO in normal saline solution (NSS), and Cur or DZG could not be detected in both the serum and tissue at each time point. The serum concentrations of Cur and DZG reached maximum concentrations at 2.28 ± 2.42 and 4.87 ± 2.74 µg/mL, respectively, within 30 min after i.p. administration. After the i.p. injection, DZG remained in the serum at detectable concentrations for up to 3 h afterwards, while concentrations of Cur declined rapidly and disappeared within 1 h (Figure 5). The tissue distribution of Cur and DZG in mouse livers, lungs and kidneys at 30 min after i.p. injection is shown in Table 2. The concentration of Cur was highest in the liver, where metabolism took place. While DZG was present at the highest concentration in the kidneys. At 1 h after i.p. injection, the DZG concentration in the kidneys increased, while the concentrations in other organs decreased (Table 2). This outcome suggests that DZG is routinely secreted out of the body via the urine. The results indicate that DZG remained in the serum at higher amounts and for longer periods than curcumin. Therefore, the deletion of β-diketone could improve the pharmacokinetics and pharmacodynamics of Cur.

## 3. Discussion

DZG, also known as feruloylmethane, is a product of the metabolic degradation of Cur that is representative of a half-structure analog of Cur. It has been found to be present in ginger as a minor active compound; therefore, its synthesis in the laboratory by the simple aldol condensation of vanillin and acetone can be considered a useful method for obtaining DZG in the in vivo study. This would enable researchers to harness its antineoplastic properties [17,18]. DZG possesses a wide range of biological activities, including antioxidant, anti-inflammation, wound healing and anticancer activities against various types of cancers, such as colon, breast, prostate and ovarian cancers [16,19]. However, the in vivo anticancer capabilities of DZG have not yet been investigated. Herein, the anticancer activity of DZG against CRPC in the in vitro study and in the PLS10 cells xenograft model in vivo and its pharmacokinetic profile were determined.

First, the anticancer activities of Cur and DZG on PLS10 cells were examined in vitro. The results indicate that Cur had a higher degree of cytotoxicity on PLS10 cells than DZG, for which the IC_50_ was found to be seven point six-fold higher than DZG (Figure 2). In agreement with the findings of previous studies, Cur displayed higher levels of in vitro cytotoxicity against lung [20] and colon cancers than DZG [16]. Our previous study showed the IC_50_ of Cur on PLS10 cells was 26.50 ± 1.80 µM, which was similar with this study [21]. This study confirmed that Cur induced G1 phase arrest via the inhibition of cyclin D1 expression in PLS10 cells, which is similar to the DZG treatment (Figure 3). Notably, in previous studies on colon cancer, HT-29 cells found that DZG in the concentration range of 250–500 µM suppressed the proliferation of HT-29 cells via induction of the cell cycle arrest in the G2/M phase [16]. Numerous published studies also reported that Cur, and its analogs, initiated cancer cell cycle arrest in both G1 and G2/M phases, depending on the type of cancer [21,22,23].Taken together, these findings suggest that DZG and Cur suppressed PLS10 cells proliferation via the inhibition of cyclin D1 expression, leading to cell cycle arrest in the G1 phase.

From our previous report, the effective anticancer dose of Cur was 30 mg/kg body weight after an i.p. injection. Therefore, in this experiment, the same concentrations of Cur and DZG were performed and compared with the efficacy in the xenograft model [21]. In addition, the DZG concentrations up to 100 mg/kg body weight showed no toxicity [24]. In the PLS10 cells xenograft model, the DZG treatment significantly (*p* < 0.05) decreased the tumor volume when compared to the control group (Figure 4a). Meanwhile, the Cur treatment at the same concentration slightly decreased the tumor volume but not significantly (*p* = 0.77). Immunohistochemistry analyses showed that the DZG treatment decreased cell proliferation in tumors but not in the Cur-treated group. Meanwhile, the apoptosis induction was increased but not significantly (*p* = 0.38) in the DZG-treated group. Both Cur and DZG significantly (*p* < 0.01) reduced the number of vessels in the tumor. Previous studies reported that Cur inhibited cell proliferation correlations with the downregulation of cyclin D1 expression in various cancer types, such as pancreatic, prostate, breast and pituitary tumor cells in both in vitro and in vivo models [25,26,27,28]. Results from both in vitro and in vivo studies could be summarized to state that DZG downregulated cyclin D1 expression, leading to the induction of the cell cycle arrest in the G1 phase, leading to a reduction in the tumor volume.

The prime obstruction of Cur for applications as a therapeutic agent is its extremely low bioavailability. After administration, Cur rapidly undergoes reduction, leading to the formation of tetrahydrocurcumin, hexahydrocurcumin and octrahydrocurcumin, or undergoes conjugation, leading to the formation of Cur-glucuronide [29]. Therefore, only small amounts of free Cur have been found in the serum or organs after administration [8,29]. To improve the bioavailability of Cur, various approaches have been used, and one of these strategies involves the use of Cur analogs [7]. With regards to DZG, a half-structure of Cur and a lack of β-diketone moiety are also considered the relevant aspects of a MAC group. The focus on Cur bioavailability and its MAC in human applications remains unclear. Our results show that DZG was found in the serum at higher concentrations and for longer periods than Cur. Cur was found to be present at high concentrations in the liver 30 min after injection but could not be detected within 1h (Figure 5). Cur was metabolized in the liver, and only free Cur was detected in this study; therefore, only a small amount of Cur was found in both the serum or tissue (Table 2). DZG was found at high concentrations in the kidneys and reached the highest concentration at 1 h after injection, suggesting that DZG might not be metabolized quite as much in the liver after administration. DZG revealed an improved level of tissue distribution than Cur, as it was found at higher concentrations and for longer periods of time than Cur (Table 2). The enhancement of the DZG bioavailability could lead to an increase in the anticancer properties of DZG in vivo. Moreover, DZG inhibited angiogenesis by a decrease in the CD31-positive area in vivo, which was similar to Cur. Taken together, this study could be summarized that the antiproliferative mechanism of DZG might be similar to Cur by the inhibition of cyclin D1 expression in PLS10 cells; however, DZG has high bioavailability in vivo than Cur, which exhibited more anticancer potential in vivo.

In our previous studies involving the cyclohexanone Cur analog, the replacement of β-diketone with the cyclohexanone group did not improve the pharmacokinetics of Cur [21]. However, in this study, we used DZG with a lack of β-diketone moiety (no other functional group replacements) and found that DZG could improve the pharmacokinetics and tissue distribution of Cur. Therefore, the deletion of β-diketone could be a new strategy for enhancing the poor bioavailability of Cur, leading to applications in treating human diseases. In addition, the DZG treatment was found to have no effect on the bodies and organ weights of mice. In another previous study, it was reported that the intraperitoneal administration of 100 mg/kg of DZG had no effect on the levels of the serum aspartate aminotransferase, alanine transaminase, serum creatinine, blood urea and lactate dehydrogenase [24]. It is noteworthy to mention that, although significant scientific progress has elucidated the anticancer activities of Cur at the molecular level in vitro, many questions and challenges still exist. Whether the anticancer properties of Cur indeed result from the presence of Cur itself or the presence of other secondary metabolites in vivo remains to be established. This study provides support for alternative applications of the Cur analog, DZG, as a safe candidate for use in clinical trials. Overall, DZG exhibited significant potential with regards to its role in the design and development of novel therapeutically active compounds with improved biological activities and pharmacokinetic properties.

## 4. Materials and Methods

### 4.1. Reagents

4-hydroxy-3-methoxybenzaldehyde (vanillin, AR grade) was purchased from Fluka Chemical AG, Buchs, Switzerland. Acetone (AR grade), sodium hydroxide (AR grade), hydrochloric acid (AR grade) and methanol (AR grade) were purchased from Labscan, Bangkok, Thailand. Potassium bromide (KBr, IR grade) was purchased from Sigma-Aldrich, St. Louis, MO, USA. Chloroform-d (CDCl_3_, purity of 99.8% D) was purchased from Cambridge Isotope Laboratories, Inc., Tewksbury, MA, USA.

### 4.2. Synthesis of Dehydrozingerone (DZG)

**Figure 6:**
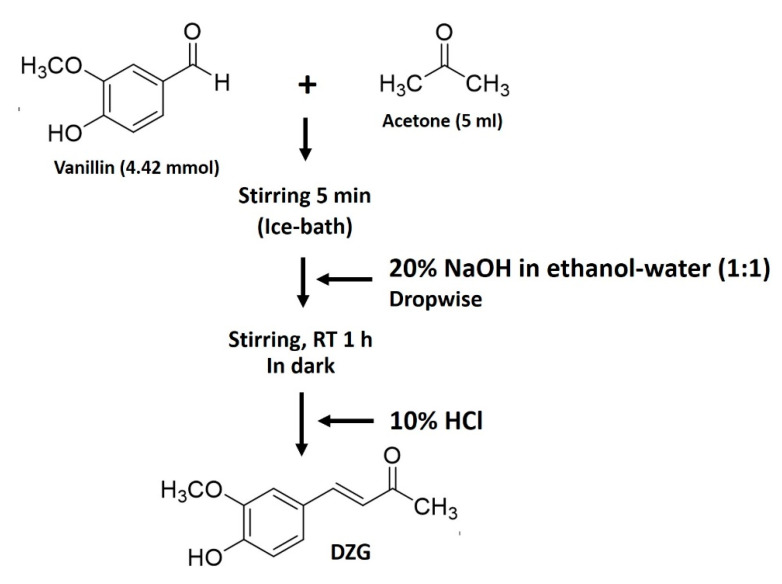
Synthesis scheme for dehydrozingerone (DZG).

DZG was synthesized via the condensation reaction (Figure 6) using a modified procedure of Ning X. et al. under the basic conditions that follow [30]. A mixture of vanillin (4-hydroxy-3-methoxybenzaldehyde) 4.42 mmol (0.68 g) in 5-mL acetone was added to a well with a closed round-bottom flask and stirred for 5 min in an ice bath. After that, 1 mL of 20% NaOH in ethanol-water (1:1) was added dropwise, and the solution was then stirred at room temperature for approximately 1 h. During the reaction period, the reactor was covered with aluminum foil to block out the light. The progress of the reaction was monitored by silica gel TLC using 3:97 methanol/dichloromethane as a developing solvent. The reaction was then worked up by adding 10% HCl solution to provide a solid product. The resulting solids were crushed into smaller pieces and filtered through filter paper under a vacuum, washed with cold water and dried in a vacuum oven. DZG was finally purified by recrystallization in ethanol to obtain a light-yellow solid.

The purity of the substance was found to be >99% by HPLC. The melting points of DZG are as follows: DZG = 157.0–159.0 °C; FT-IR (ν, cm^−1^ in KBr) 3281.83 (O-H stretch, phenol), 3001.76–2848.99 (C-H stretch), 1636.63 (C=O stretch, conjugate ketone), 1582.62, 1518.22 (C=C stretch in aromatic ring), 1265.07, 1025.83 (C-O stretch, phenol, alkoxy) and 823.09 (=C-H bending of trisubstituted alkene). ^1^H-NMR *δ* (ppm, 500 MHz, CDCl_3_) 7.42 (1H, *d*, *J_trans_* = 16.20 Hz), 7.06 (1H, *dd*, *J_ortho_*_, *meta*_ = 8.13, 1.87 Hz), 7.03 (1H, *d*, *J_meta_* = 1.83 Hz), 6.91 (1H, *d*, *J_ortho_* = 8.16 Hz), 6.56 (1H, *d*, *J_trans_* = 16.20 Hz), 6.05 (1H, *br*), 3.90 (3H, *s*) and 2.34 (3H, *s*); ^13^C-NMR *δ* (ppm, 125 MHz, CDCl_3_) 198.57, 148.26, 146.86, 143.85, 129.27, 126.74, 124.80, 123.46, 114.80, 55.86 and 27.20.

### 4.3. Cell Culture

Rat CRPC PLS10 cells were effectively established in our laboratory [31,32]. The cells were cultured in Roswell Park Memorial Institute-1640 medium (RPMI 1640, Gibco, Carlsbad, CA, USA) with 10% fetal bovine serum (FBS, Thermo Scientific Company, Waltham, MA, USA) ), 5-mM L-glutamine, 50-IU/mL penicillin and 50-mg/mL streptomycin and maintained in a humidified incubator with an atmosphere comprised of 95% air and 5% CO_2_ at 37°C. When the cells reached 70–80% confluence, they were harvested and plated for subsequent passages or for Cur or DZG treatments.

### 4.4. Animals

All experimental animals were housed under protocols approved by the Institutional Animal Care and Use Committee of Nagoya City University Graduate School of Medical Sciences, Nagoya, Japan. Seven-week-old male BALB/c mice and five-week-old male BALB/c-nu/nu mice were purchased from Charles River Japan, Inc. (Atsugi, Japan) and housed in plastic cages with hardwood chip bedding in an air-conditioned room at 23 ± 2°C and at 55% ± 5% humidity with a 12-h light/dark cycle. A basal animal diet (Oriental MF, Oriental Yeast Co., Tokyo, Japan) and distilled water were made available ad libitum. Subjects were used in this study after a single week of the acclimation period.

### 4.5. Cell Viability Assay

PLS10 cells (2.0 × 10^3^ cells per well) were plated in a 96-well plate for 24 h. Cur and DZG were dissolved in DMSO and diluted to the final concentration in the culture medium. After that, the culture medium containing Cur (0–50 µM) or DZG (0–200 µM) in 0.5% DMSO (vehicle control) were added into the cells. The cells were incubated in 5% CO_2_ at 37°C for a further 48 h, and cell viability was determined using the WST-1 colorimetric assay (Roche Diagnostics, Mannheim, Germany). In each experiment, determinations were carried out in triplicate.

### 4.6. Cell Cycle Analysis

The PLS10 cells (1.0 × 10^5^ cells per well) were plated into a 6-well plate overnight. Then, cells were treated with increasing concentrations of Cur (0–20 µM) or DZG (0–200 µM) in the present of 0.5% DMSO (vehicle control) for the next 48 h. Cell morphology was investigated and photographed under a microscope. The cell cycle distribution was determined using a Guava cycle assay (Guava Technologies, Hayward, CA, USA) according to the manufacturer’s protocols. Briefly, the cells were removed by trypsinization, and the cell suspension was fixed in ice-cold 70% methanol at −20°C. After that, the methanol was removed, and the cells were washed with PBS before being stained with a Guava cycle reagent for 30 min. Cell cycle distribution was determined on a Guava PCA Instrument using Guava®ViacountTM Software (Merck Ltd., Darmstadt, Germany).

### 4.7. Western Blot Analysis

Protein concentration was determined using the Bradford method (Thermo Fisher Scientific, Waltham, MA, USA). For the examination of protein expression levels in lysed cells, the proteins were separated by SDS-PAGE using 10% acrylamide gel. Then, the separated proteins were transferred onto a nitrocellulose membrane and blocked with 5% (*w*/*v*) skim milk in 1 × TBS containing 0.3% (*v*/*v*) Tween (TBS-T) at room temperature for 1 h. Membranes were washed twice with TBS-T and incubated with the primary antibody, mouse anti-cyclin D1 (Merck Millipore, Danvers, MA, USA) at 4°C overnight. After being washed five times with TBS-T, membranes were incubated with the horseradish peroxidase (HRP)-conjugated anti-mouse IgG (GE Healthcare Biosciences, Buckinghamshire, NA, UK) at room temperature for 2 h and then washed with TBS-T five times. Immunoreactive material was then visualized with an enhanced chemiluminescence detection system (GE Healthcare Biosciences). To confirm equal protein loading, each membrane was stripped and incubated with mouse anti-β-actin (Sigma-Aldrich).

### 4.8. In Vivo Antitumor Activity

The in vivo antitumor activity of DZG or Cur was examined using five-week-old male BALB/c-nu/nu mice. Mice were randomly divided into 3 groups, with up to 8 or 9 mice per group. The PLS10 cells (5.0 × 10^5^) were subcutaneously injected into each mouse. The treatments were initiated 2 days after PLS10 cells injections, at which point, mice received 50% DMSO in normal saline solution (NSS) (vehicle control) at 30 mg/kg body weight of Cur or DZG via i.p. injection two times a week for five weeks. The body weight of each mouse was measured twice a week during the experiment. At week 5 of the experiment, mice were euthanized, and primary tumors and organs, such as lungs, liver, kidneys and lymph nodes, were removed and fixed in 10% buffered formalin. The tumor volume of each mouse was measured and calculated according to the following formulation: 0.52 × (axis1 × axis2 × axis3) [11,33]. A section of the primary tumor, lung, liver, and kidney specimens were collected and fixed in 10% buffered formalin. Tissue sections of the primary tumors and organs were stained with H&E.

### 4.9. Immunohistochemistry

The paraffin-embedded samples were sectioned and stained with rabbit monoclonal anti-Ki67 (SP6; Acris Antibodies GmbH, Herford, Germany) and rabbit polyclonal anti-CD31 antibodies (Abcam, Cambridge, UK) and sequentially stained with an anti-rabbit secondary antibody and avidin-biotin complex. The binding complexes were then visualized with diamino-benzidine. Subsequently, the sections were then counterstained lightly with hematoxylin for microscopic examination. The number of Ki67-staining cells for a minimum of 1000 cells was counted to determine the labeling index. For quantitative analysis of the vessel area, CD31-positive areas per the total area section were determined under microscopy (BZ-9000).

### 4.10. Terminal Deoxynucleotidyl Transferase dUTP Nick end Labeling (TUNEL) Assay

The paraffin-embedded samples were sectioned, and the degree of apoptosis in the tumor tissue was determined using a TUNEL assay (In situ Apoptosis Detection Kit; Takara Bio Inc., Ohtsu, Japan). The TUNEL assay was performed according to the manufacturer’s instructions. The number of TUNEL-labeled cells in a minimum of 1000 cells was counted in order to establish the labeling index.

### 4.11. In Vivo Pharmacokinetics and Tissue Distributions

A comparison of the pharmacokinetics between Cur and DZG was determined using seven-week-old male BALB/c mice. The designated 32 mice were randomly divided into 3 groups, identified as the control, Cur and DZG groups. Mice were i.p. injected with 50% DMSO in NSS (vehicle control), Cur or DZG at concentrations of 100 mg/kg of body weight for each mouse. The mice were euthanized, and blood from each mouse was collected at 0, 30, 60 and 180 min after injection. Lungs, liver and kidneys were removed and frozen for detection of the levels of concentrations of Cur and DZG.

### 4.12. Serum and Tissue Concentrations of Cur and DZG

The concentrations of Cur or DZG in the serum or organ tissues were determined using the HPLC method. The tissues were homogenized in 200 μl of 50% acetonitrile and vortexed for 5 min. Samples were centrifuged at 96.5 g at 4°C for 20 min. Fifty microliters of serum or tissue homogenate was mixed with 1.5-mg/mL trichloroacetic acid in 100 µl of acetonitrile. The substance was then centrifuged at 96.5 g at 4°C for 20 min. Sixty microliters of the supernatant was collected and subjected to HPLC using a reversed-phase C18 column (Waters Corporation, Milford, MA, USA). The mobile phase was composed of 52% acetonitrile and 48% citric buffer (1% w/v citric acid, pH 3.0), and the flow rate was set at 1 mL/min. The detection wavelength for Cur and DZG were 425 and 340 nm, respectively [11,34]. Concentrations of Cur and DZG in the serum were calculated and compared to the standard curve (0–3200 ng/mL). The limits of quantification for Cur and DZG were 100 and 50 ng/mL, respectively. The extraction recovery from the plasma was approximately 90% for both Cur and DZG.

### 4.13. Statistical Analysis

In the in vivo experiment, the data are presented as mean ± standard error of the mean (S.E.) values, while the data from the in vitro experiment were collected from experiments conducted in triplicate to confirm reproducibility and were then presented as mean ± standard deviation (S.D.) values. Statistical analysis was performed with Prism version 6.0 software (GraphPad Software, San Diego, CA, USA) using a one-way ANOVA, Dunnett’s multiple comparison test, with the degree of significance being determined at *p < 0.05, ** *p* < 0.01 and *** *p* < 0.001.

## 5. Conclusions

Our data from the in vitro study revealed that Cur displayed more potent anticancer capabilities than DZG. However, in the in vivo study, DZG was found to significantly inhibit tumor growth, whereas Cur did not. The anticancer capability of DZG might be due to the reduction in cell proliferation that occurs by inhibiting cyclin D1 expression and angiogenesis. Moreover, DZG displayed higher in vivo pharmacokinetics and pharmacodynamics than Cur. Taken together, these findings indicate that DZG may serve as an attractive molecule with various beneficial clinical applications in the near future.

## Figures and Tables

**Table 1:** The body weights or organ weights of mice at the end of the experiment.

Treatment Groups	Body Weights	Organ Weights	
		Livers	Kidneys
**Control**	26.75 ± 1.83	1.59 ± 0.20	0.40 ± 0.05
**Cur (30 mg/kg)**	26.71 ± 1.26	1.67 ± 0.10	0.40 ± 0.03
**DZG (30 mg/kg)**	27.12 ± 1.00	1.60 ± 0.09	0.39 ± 0.03

Values are mean ± S.E. of eight mice in the control and nine mice in the treatment groups. Cur: curcumin and DZG: dehydrozingerone.

**Table 2:** Tissue distribution of Cur and DZG in mice after receiving a single dose of Cur or DZG.

Tissue	30 min		60 min		180 min	
	Cur (µg/g)	DZG (µg/g)	Cur (µg/g)	DZG (µg/g)	Cur (µg/g)	DZG (µg/g)
Liver	2.60 ± 0.78	0.92 ± 0.67	ND	0.41 ± 0.11	ND	ND
Kidneys	1.01 ± 0.55	5.92 ± 3.80	0.53 ± 0.19	9.75 ± 4.08	ND	0.86 ± 0.22
Lung	1.15 ± 1.30	1.41 ± 0.74	ND	0.72 ± 0.28	ND	ND

ND = not detectable. Cur or DZG (100 mg/kg body weight) was administered to each mouse. The mice were sacrificed at 30, 60 and 180 min after injection. Values are expressed as mean ± S.E. of three or four mice.
